# Luspatercept for myelodysplastic syndromes/myeloproliferative neoplasm with ring sideroblasts and thrombocytosis

**DOI:** 10.1038/s41375-022-01521-4

**Published:** 2022-02-26

**Authors:** Rami S. Komrokji, Uwe Platzbecker, Pierre Fenaux, Amer M. Zeidan, Guillermo Garcia-Manero, Ghulam J. Mufti, Valeria Santini, María Díez-Campelo, Carlo Finelli, Joseph G. Jurcic, Peter L. Greenberg, Mikkael A. Sekeres, Amy E. DeZern, Michael R. Savona, Jeevan K. Shetty, Rodrigo Ito, George Zhang, Xianwei Ha, Jay T. Backstrom, Amit Verma

**Affiliations:** 1 Moffitt Cancer Center, Tampa, FL USA; 2 Medical Clinic and Policlinic 1, Hematology and Cellular Therapy, University Hospital Leipzig, Leipzig, Germany; 3 Service d’Hématologie Séniors, Hôpital Saint-Louis, Assistance Publique-Hôpitaux de Paris and Université Paris 7, Paris, France; 4 Department of Internal Medicine, Yale School of Medicine and Yale Cancer Center, Yale University, New Haven, CT USA; 5 Department of Leukemia, The University of Texas MD Anderson Cancer Center, Houston, TX USA; 6 Department of Haemato-Oncology, King’s College London, London, UK; 7 MDS Unit, AOU Careggi, University of Florence, Florence, Italy; 8 Hematology Department, Institute of Biomedical Research of Salamanca, University Hospital of Salamanca, Salamanca, Spain; 9 IRCCS Azienda Ospedaliero-Universitaria di Bologna, Institute of Hematology “Seràgnoli”, Bologna, Italy; 10 Division of Hematology/Oncology, Herbert Irving Comprehensive Cancer Center, Columbia University Medical Center, New York, NY USA; 11 Stanford University Cancer Center, Stanford, CA USA; 12 Division of Hematology, Sylvester Cancer Center, University of Miami, Miami, FL USA; 13 The Sidney Kimmel Comprehensive Cancer Center at Johns Hopkins, Baltimore, MD USA; 14 Vanderbilt-Ingram Cancer Center, Vanderbilt University School of Medicine, Nashville, TN USA; 15 Celgene International Sàrl, a Bristol-Myers Squibb Company, Boudry, Switzerland; 16 Bristol Myers Squibb, Princeton, NJ USA; 17 Acceleron Pharma, Cambridge, MA USA; 18 Department of Oncology, Albert Einstein College of Medicine, Montefiore Medical Center, New York, NY USA

**Keywords:** Randomized controlled trials, Myelodysplastic syndrome

## To the Editor:

Myelodysplastic syndromes/myeloproliferative neoplasm with ring sideroblasts and thrombocytosis (MDS/MPN-RS-T) is a myeloid disorder with myelodysplastic and myeloproliferative features [1, 2]. MDS/MPN-RS-T-associated anemia causes fatigue, reduced quality of life, and worse survival [3–5]. Patients with MDS/MPN-RS-T have favorable overall survival compared to patients with MDS-RS [6]; however, ~50% of patients require red blood cell (RBC) transfusions resulting in protracted transfusion dependence. Patients with MDS/MPN-RS-T also have a fourfold higher thrombotic event risk compared to patients with MDS with ring sideroblasts (MDS-RS) [7]. Treatment of MDS/MPN-RS-T aims to improve anemia, reduce thrombotic event risk, lower platelets, and/or modify the disease course [6, 8]. However, data supporting the efficacy of these treatments are scarce.

Luspatercept is a first-in-class erythroid maturation agent that binds several transforming growth factor-β superfamily ligands, enhancing late-stage erythropoiesis [9]. The results of the phase 3 MEDALIST study (NCT02631070) [10] led to its approval by the US Food and Drug Administration and the European Medicines Agency for the treatment of anemia in adults with lower-risk MDS-RS or MDS/MPN-RS-T requiring ≥2 RBC units/8 weeks after erythropoiesis-stimulating agent (ESA) failure [11]. Here, we report a post hoc analysis of luspatercept efficacy and safety in patients with MDS/MPN-RS-T from the MEDALIST study. The MEDALIST study enrolled 229 adults with lower-risk MDS-RS who required ≥2 RBC units/8 weeks and were refractory or intolerant to ESAs [10]. Patients were randomized 2:1 to luspatercept or placebo, administered subcutaneously every 3 weeks for 24 weeks. Luspatercept starting dose was 1.0 mg/kg, with titration to a maximum of 1.75 mg/kg, according to transfusion requirements and adverse events [10]. The diagnosis of patients with MDS/MPN-RS-T in the intention-to-treat population was performed using cytomorphologic, cytogenetic, and molecular genetic results and blood counts.

**Fig. 1:**
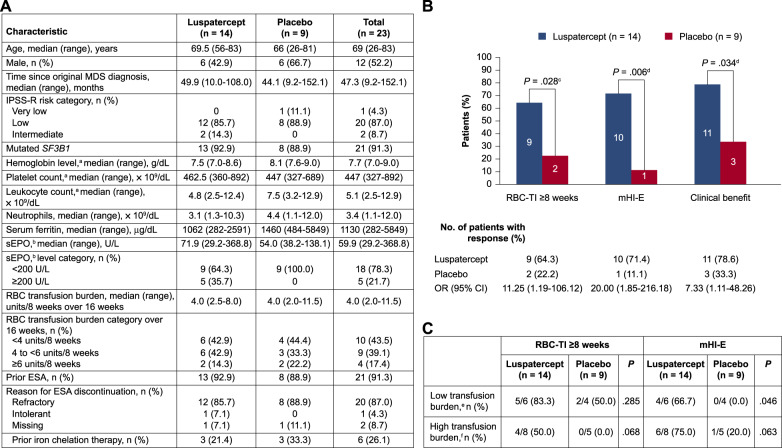
Baseline characteristics and treatment response of patients with MDS/MPN-RS-T in the MEDALIST trial. Baseline characteristics (**A**). Rates of RBC-TI ≥8 weeks, mHI-E, and clinical benefit during weeks 1–24 (**B**). Response during weeks 1–24 by baseline transfusion burden (**C**). CI confidence interval, CMH Cochran-Mantel-Haenszel, ESA erythropoiesis-stimulating agent, IPSS-R Revised International Prognostic Scoring System, MDS myelodysplastic syndromes, MDS/MPN-RS-T myelodysplastic syndromes/myeloproliferative neoplasm with ring sideroblasts and thrombocytosis, mHI-E modified hematologic improvement–erythroid, OR odds ratio, RBC red blood cell, RBC-TI red blood cell transfusion independence, sEPO serum erythropoietin. ^a^Last value measured on or before the date and time of the first dose of luspatercept/placebo. ^b^Highest value within 35 days before the first dose of luspatercept/placebo. ^c^CMH test stratified for average baseline RBC transfusion requirement (≥6 vs <6 units of RBC per 8 weeks) and baseline IPSS-R score (Very low or Low vs Intermediate). ^d^Un-stratified CMH test. ^e^Defined as baseline transfusion burden <4 RBC units/8 weeks. ^f^Defined as baseline transfusion burden ≥4 RBC units/8 weeks.

Of the 229 patients in MEDALIST study, 23 (10.0%) had MDS/MPN-RS-T; 14 were randomized to luspatercept and 9 to placebo. Baseline characteristics that differed between the two arms included lower median leukocyte count (4.8 vs 7.5 × 10^9^/dL) and serum ferritin (1062 vs 1460 µg/dL), and higher serum erythropoietin (sEPO) (71.9 vs 54.0 U/L) (Fig. 1A). Median (range) follow-up times were 27.4 (3.5–35.6) and 13.8 (3.3–32.2) months in the luspatercept and placebo arms, respectively.

The primary endpoint in the MEDALIST study was RBC transfusion independence (RBC-TI) ≥8 weeks during weeks 1–24. Secondary endpoints included: modified hematologic response–erythroid (mHI-E; mean hemoglobin increase ≥1.5 g/dL [patients receiving <4 RBC units/8 weeks at baseline] or a reduction of ≥4 units RBC transfusion [patients receiving ≥4 RBC units/8 weeks at baseline], over 56 consecutive days) [12]; ≥1.0 g/dL hemoglobin increase from baseline over 56 consecutive days during weeks 1–24; rates of progression to acute myeloid leukemia (AML); and incidence of treatment-emergent adverse events (TEAEs). A post hoc analysis of clinical benefit (defined as RBC-TI ≥8 weeks and/or mHI-E during weeks 1–24) was also performed. All *P* values are descriptive and not adjusted for multiplicity.

**Fig. 2:**
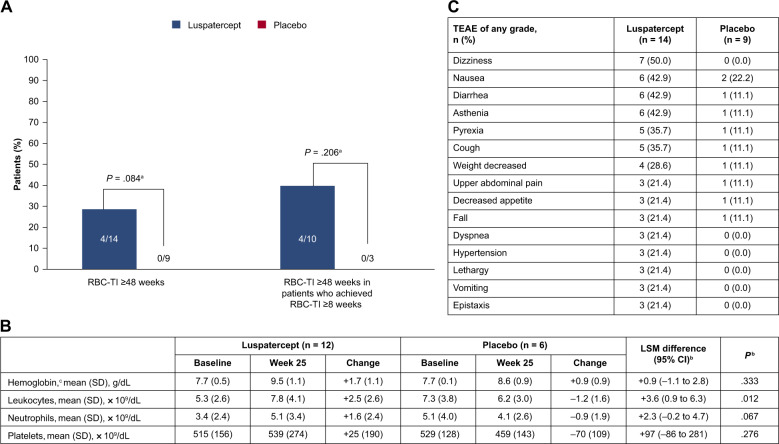
Treatment response, laboratory parameters, and TEAEs. Rates of RBC-TI ≥48 weeks at any time during treatment in all patients with MDS/MPN-RS-T and in those with RBC-TI ≥8 weeks at any time while on treatment (**A**). Laboratory parameters at baseline and at week 25 of treatment (**B**). TEAEs of any grade occurring in ≥20% of patients in either group (**C**). ANCOVA analysis of covariance, CI confidence interval, CMH Cochran-Mantel-Haenszel, LSM least-squares mean, MDS/MPN-RS-T myelodysplastic syndromes/myeloproliferative neoplasm with ring sideroblasts and thrombocytosis, RBC-TI red blood cell transfusion independence, SD standard deviation, TEAE treatment-emergent adverse event. ^a^Un-stratified CMH test. ^b^Estimates are based on an ANCOVA model with treatment (luspatercept vs placebo) as main fact, and baseline value as a covariate (for neutrophils, baseline leukocytes were used as a covariate). ^c^Data are only available for six patients in the luspatercept group and three in the placebo group.

As of July 2019, a significantly higher proportion of patients with MDS/MPN-RS-T randomized to luspatercept achieved RBC-TI ≥8 weeks during weeks 1–24 (64.3 vs 22.2%; *P* = 0.028); mHI-E (71.4 vs 11.1%; *P* = 0.006); and clinical benefit (78.6 vs 33.3%; *P* = 0.034), vs placebo (Fig. 1B). The median (range) time from clinical benefit start to the end of treatment was 94.6 (range 8.0–150.0) weeks in the luspatercept arm and 23.9 (range 23.7–57.9) weeks with placebo. A numerically higher number of low transfusion burden patients (<4 units/8 weeks) randomized to luspatercept vs placebo achieved RBC-TI ≥8 weeks (5/6 [83.3%] vs 2/4 [50.0%]; *P* = 0.285) during weeks 1–24, and a significantly greater proportion achieved mHI-E (4/6 [66.7%] vs 0/4 [0.0%]; *P* = 0.046) (Fig. 1C). A numerically higher number of high transfusion burden patients (≥4 units/8 weeks) randomized to luspatercept vs placebo achieved RBC-TI ≥8 weeks (4/8 [50.0%] vs 0/5 [0.0%]; *P* = 0.068) and mHI-E (6/8 [75.0%] vs 1/5 [20.0%]; *P* = 0.063) (Fig. 1C). RBC-TI ≥8 weeks during weeks 1–48 was achieved by 64.3% of patients randomized to luspatercept vs 33.3% for placebo (*P* = 0.088). RBC-TI ≥48 weeks at any time during treatment was achieved by 28.6% of patients randomized to luspatercept vs no placebo patients; among those patients in the luspatercept group who had reached RBC-TI ≥8 weeks at any time during treatment, 40.0% achieved RBC-TI ≥48 weeks, vs no placebo patients (Fig. 2A).

Despite limited numbers, the value of luspatercept for patients with MDS/MPN-RS-T is supported by comparisons with data from the entire MEDALIST study population. The achievement of RBC-TI ≥8 weeks during weeks 1–24 among patients with MDS/MPN-RS-T randomized to luspatercept vs placebo (64.3 vs 22.2%) was higher than in the overall MEDALIST population (37.9 vs 13.2%) [10]. Similarly, mHI-E was achieved in 71.4 vs 11.1% of patients with MDS/MPN-RS-T randomized to luspatercept vs placebo, compared to 52.9 vs 11.8% in the overall MEDALIST population [10].

After 24 weeks of treatment, patients randomized to luspatercept had increases from baseline in mean [standard deviation, SD] hemoglobin (7.7 [0.5]–9.5 [1.1] g/dL), leukocytes (5.3 [2.6]–7.8 [4.1] × 10^9^/dL), and neutrophils (3.4 [2.4]–5.1 [3.4] × 10^9^/dL), while platelet levels remained stable (515 [156] and 539 [274] × 10^9^/dL) (Fig. 2B). Although the increase in hemoglobin levels after 24 weeks among patients with MDS/MPN-RS-T was not significantly different between luspatercept and placebo, the absolute magnitude of increase was nominally higher (+1.7 vs +0.9 g/dL) [10]. Patients randomized to luspatercept vs placebo had a significantly greater increase in mean leukocyte count but not mean platelet or neutrophil counts. At baseline, patients with MDS/MPN-RS-T had a higher median platelet count than the overall MEDALIST population (447 vs 234 × 10^9^/dL) as expected, had lower median sEPO (59.9 vs 153.2 U/L), were less likely to have received iron chelation therapy (26.1 vs 48.5%), and had lower median transfusion burden (4.0 vs 5.0 units/8 weeks), consistent with their higher RBC-TI and mHI-E response rates [10].

The most common TEAEs of any grade in the luspatercept arm were dizziness, nausea, diarrhea, and asthenia (Fig. 2C). TEAEs leading to discontinuation occurred in 2 of 14 (14.3%) patients in the luspatercept arm and 3 of 9 (33.3%) in the placebo arm. One patient randomized to luspatercept experienced a transient ischemic attack. One patient randomized to placebo experienced progression to AML (*P* = 0.202) vs none randomized to luspatercept.

Recommendations for the treatment of patients with MDS/MPN-RS-T include ESAs and transfusions for anemia, and lenalidomide for anemia and platelet-level reduction [7, 8]. Recommendations for the use of lenalidomide for patients with MDS/MPN-RS-T are based on case reports totaling 12 patients [13] and a retrospective analysis of 167 patients [14], rather than clinical trials; the use of ESAs is based on a single retrospective study which included 40 patients with MDS/MPN-RS-T, of whom 45% achieved an erythroid response (hemoglobin increase ≥2.0 g/dL or RBC-TI ≥8 weeks for patients who required ≥4 units/8 weeks) [15], compared to 71.4% of patients treated with luspatercept (refractory or ineligible for ESAs) in the current study. However, this comparison should be undertaken with caution, given the different definitions of erythroid response.

In conclusion, this subgroup analysis provides the first clinical trial data to support the efficacy of luspatercept in patients with MDS/MPN-RS-T, a population who currently have no proven effective treatment options. Overall, luspatercept was found to be effective—significantly reducing transfusion burden and improving mHI-E and leukocyte levels—with a generally well-tolerated safety profile.

## References

[1] Arber DA, Orazi A, Hasserjian R, Thiele J, Borowitz MJ, Le Beau MM (2016). The 2016 revision to the World Health Organization classification of myeloid neoplasms and acute leukemia. Blood.

[2] Patnaik MM, Lasho T (2020). Myelodysplastic syndrome/myeloproliferative neoplasm overlap syndromes: a focused review. Hematol Am Soc Hematol Educ Program..

[3] Patnaik MM, Lasho TL, Finke CM, Hanson CA, King RL, Ketterling RP (2016). Predictors of survival in refractory anemia with ring sideroblasts and thrombocytosis (RARS-T) and the role of next-generation sequencing. Am J Hematol.

[4] Stauder R, Valent P, Theurl I (2018). Anemia at older age: etiologies, clinical implications, and management. Blood.

[5] Germing U, Oliva EN, Hiwase D, Almeida A (2019). Treatment of anemia in transfusion-dependent and non-transfusion-dependent lower-risk MDS: current and emerging strategies. [Abstract]. Hemasphere.

[6] Patnaik MM, Tefferi A (2021). Myelodysplastic syndromes with ring sideroblasts (MDS-RS) and MDS/myeloproliferative neoplasm with RS and thrombocytosis (MDS/MPN-RS-T) - “2021 update on diagnosis, risk-stratification, and management”. Am J Hematol..

[7] Broseus J, Florensa L, Zipperer E, Schnittger S, Malcovati L, Richebourg S (2012). Clinical features and course of refractory anemia with ring sideroblasts associated with marked thrombocytosis. Haematologica.

[8] National Comprehensive Cancer Network (NCCN). NCCN Clinical Practice Guidelines in Oncology (NCCN Guidelines). Myelodysplastic syndromes. Version 3.2021. 2021. https://www.nccn.org/professionals/physician_gls/pdf/mds.pdf. Accessed 3 Nov 2021.

[9] Attie KM, Allison MJ, McClure T, Boyd IE, Wilson DM, Pearsall AE (2014). A phase 1 study of ACE-536, a regulator of erythroid differentiation, in healthy volunteers. Am J Hematol.

[10] Fenaux P, Platzbecker U, Mufti GJ, Garcia-Manero G, Buckstein R, Santini V (2020). Luspatercept in patients with lower-risk myelodysplastic syndromes. N Engl J Med..

[11] US Food and Drug Administration (FDA). FDA approves luspatercept-aamt for anemia in adults with MDS. 2020. https://www.fda.gov/drugs/resources-information-approved-drugs/fda-approves-luspatercept-aamt-anemia-adults-mds. Accessed 3 Nov 2021.

[12] Cheson BD, Greenberg PL, Bennett JM, Lowenberg B, Wijermans PW, Nimer SD (2006). Clinical application and proposal for modification of the International Working Group (IWG) response criteria in myelodysplasia. Blood.

[13] Divoux M, Plocque A, Sevin M, Voillat L, Feugier P, Guerci-Bresler A (2020). Efficacy of lenalidomide in myelodysplastic/myeloproliferative neoplasms with ring sideroblasts and an extreme platelet count. Clin Case Rep..

[14] Komrokji R, Melody M, Al Ali N, Chan O, Klimek V, Ball BJ, et al. Treatment outcomes for patients with myelodysplastic syndrome/myeloproliferative neoplasms with ring sideroblasts and thrombocytosis. Leuk Lymphoma. 2021. 10.1080/10428194.2021.1971217. 10.1080/10428194.2021.1971217 34448437

[15] Antelo G, Mangaonkar AA, Coltro G, Buradkar A, Lasho TL, Finke C (2020). Response to erythropoiesis-stimulating agents in patients with WHO-defined myelodysplastic syndrome/myeloproliferative neoplasm with ring sideroblasts and thrombocytosis (MDS/MPN-RS-T). Br J Haematol..

